# Socio-Moral Development of Preschool Children: Aspects of Theory and Practice

**DOI:** 10.3390/bs9120129

**Published:** 2019-11-27

**Authors:** Evgeniya Korotaeva, Irina Chugaeva

**Affiliations:** Institute of Pedagogy and Psychology of Childhood, Ural State Pedagogical University, 26, Cosmonauts Ave, 620017 Yekaterinburg, Russia; irinachugaeva555@mail.ru

**Keywords:** social and moral education, preschool age, communitarian approach

## Abstract

This article deals with issues relating to the social and moral education and development of preschool children. The theoretical understanding of the current state of this field is reflected in the modern normative documents “On education in the Russian Federation”, the state educational standards. The analysis of theoretical and practice-oriented research in this area shows that the phrase “moral education” today is gradually being replaced by “social and moral education” and “social and moral development”. This trend is found in preschool pedagogy. It is the study of social and moral representations of preschool children that many relevant diagnostic methods are aimed at: to explain the actions of children and their relationships with each other and adults, and to assess these actions (i.e., to correlate the situation with the moral norm). The presented research was based on the method of research of moral representations of children of senior preschool age. The analysis of the results showed that children of senior preschool age willingly include themselves in the retelling of the actions described in situations but find it difficult to assess what is happening from a moral point of view. It is obvious that psychological and pedagogical work is necessary in this direction, taking into account the age characteristics of the children, as well as the social and moral guidelines relevant to the current situation in regard to the development of society.

## 1. Introduction

Issues relating to the moral education of the younger generation have always been a priority for pedagogy. To the questions of the formation of morality, one way or another, these were addressed by such outstanding educators as J.A Comenius [1], Zh.-Zh Russo. [2], L. N. Tolstoy [3].

In understanding the foundations of moral education, four traditions are revealed: paternalistic (assuming as an obligatory component the veneration of elders); religious and ecclesiastical (based on the maintenance of the authority of faith and the Church); educational (including the active development of scientific knowledge, subject to the judgment of the mind) and communitarian (based on the idea of social order, which contributes to the development of community ties and the formation of collectivism).

Significant social changes that occurred in the late XX-early XXI centuries—globalization, mass informatization, openness, the destruction of the old ideology, etc.—have led to the fact that today a communitarian approach occupies the priority position in moral education. This is evident even in the leading terms and concepts used in pedagogical theory and practice. The Federal law “On education in the Russian Federation” (2012) focused on spiritual and moral development, improvement, spiritual and moral culture and relevant values [4]. This, of course, was influenced by the “Concept of spiritual and moral development and education of the individual of a citizen of Russia” published in 2009 [5].

Today, the phrase “moral education” is gradually being replaced by “social and moral education” and “social and moral development”. This substitution emphasizes that morality is understood not simply as a set of rules that determines the behavior of one person, but as a certain mechanism that regulates the existence of a particular society. Morality is defined as the process of interactions between individuals and groups of individuals, through their relation to social phenomena and life in general [6,7,8].

Let us note that the example of educational programs for preschool education does not use the phrase “spiritual and moral”, but it is designated as “social and moral development and education” [9,10,11,12,13].

It is not by chance that the diagnostic methods that allow us to draw a conclusion about the level of moral development of a preschool child are based on the understanding of situations of communication and contact with peers and adults. And this is also seen as the basis of the communitarian approach in the educational process. Empirical studies of the socio-moral development of preschoolers help to identify the synergy of the interaction of the subjects of the educational process, to review the activities of the subjects through the determination of social communities, social institutions, and social consciousness in general.

This trend is also found in preschool pedagogy. Thus, the Federal state requirements for the structure of the basic general education program of preschool education since 2010 have designated one educational area “socialization”, which had the most common name and was in fourth place in the list of ten educational areas [14]. But in the Federal state educational standards of preschool education in 2013, the name “social and communicative development” and its place, which headed the list of five educational areas, also changed. The content of this area is revealed through moral and ethical values, the development of social and emotional intelligence, emotional responsiveness, empathy, the formation of respect and a sense of belonging to his or her family and to the community of children and adults in the preschool organization [15].

It is obvious that this change could not but be reflected in the relevant programs designed for the practice of preschool education. For example, “A Typical program of training and education in kindergarten” [16] was overly ideologized, while the section on the moral education of preschool children was developed in insufficient detail and did not correspond to the realities of social life. At the same time, in the approximate program “From birth to school” [17], the area of social and communicative development of a preschool child was more clearly structured and decomposed:

- assimilation of norms and values adopted in society, education of moral and ethical qualities of the child, the formation of the ability to correctly assess their actions and actions of peers;
- formation of children’s readiness for joint activities, development of the ability to negotiate, independently resolve conflicts with peers;
- development of emotional responsiveness, empathy, respectful and friendly attitude to others;
- formation of the image of I in the family, in the organization, children and adult communities, etc.

It is obvious that today it is becoming more than an urgent task for a preschool child to gain experience of moral behavior in social communication on the basis of their understanding of personal traits as a result of the experience of interaction with adults and peers, experiences of social feelings (respect, responsibility, involvement, etc.), and the ability to evaluate their actions and actions of peers from the standpoint of the well-being of all.

In view of the above, there is a need for research that can reveal common approaches and certain aspects in the process of the formation of moral norms in preschool children in various social situations.

We set a task to identify the features of the assimilation of social norms and rules of morality in children of senior preschool age attending kindergarten, and to clarify the possibility of their social and moral development.

## 2. Materials and Methods

The theoretical basis was the existential approach in the study of social behavior of a person. We proceeded from the statement that social behavior is characterized by duality: on the one hand, a person’s actions are determined by social standards and norms; on the other hand, actions are controlled by the person and his/her ability to choose and bear responsibility for this choice [18]. In the framework of the existential approach, a preschool child is treated as a subject to satisfy their needs, interests and aspirations. In childhood, a child gets experience of social relations, assimilates moral standards through personal perception and learns to solve problems. Experiments of free choice help to reveal the presence of personal judgments, disagreements, and suggestions in children and to raise their socio-philosophical sensitivity [19]. To identify ideas in preschoolers about the social norm, as a rule, we refer to the projective techniques in which the child has the opportunity to choose his or her own solutions to any situation. We addressed the diagnostic method of G. A. Uruntaeva and Yu.A. Afon’kina [20] in which children were offered four problem situations of communication and interaction. Each situation contained a problem of a choice of an act (to share a pencil, a doll, to take (or to evade) responsibility for the broken toy, etc.), and also motivation to give an explanation of motives of the chosen action [20] (pp. 59–61). Each situation contained a task to choose an action and a stimulus to explain the motives of the chosen action [20].

Presentation of the task: in an individual conversation with a child in the kindergarten group, he or she was asked to continue each of the given stories and answer the questions. The child was told: “I will tell you stories, finish them”. After that, four stories were read to the child one after another (in any order).

Situation 1: Lyuba and Sasha were drawing. Lyuba used a red pencil and Sasha used a green one. Suddenly Lyuba’s pencil broke. “Sasha”, said Lyuba, “May I take your pencil to finish my picture?” Sasha answered... What did Sasha answer? Why? What did Sasha do? Why?

Situation 2: Katya’s mother gave her a beautiful doll for her birthday. Katya began to play with it. Her younger sister Vera came up to her and said, “I want to play with this doll too.” Then Katya answered... What did Katya say? Why? What did Katya do? Why?

Situation 3: Children were building a city. Olya was standing nearby watching the children play. The teacher came up to the children and said, “We are going to have dinner now. It’s time to put the cubes in the box. Ask Olya to help you”. And Olya answered... What did she say? Why? What did Olya do? Why?

Situation 4: Petya and Vova were playing together and broke a beautiful and expensive toy. Their Dad came and asked, “Who broke the toy?”. Petya answered... What did he say? Why? What did Petya do? Why?

## 3. Results

This experiment was carried out among preschool children in one of the groups in Yekaterinburg kindergarten. According to the requirements of the Federal state educational standards, the parents of these children agreed to the participation of their children in these studies.

The quantitative analysis of the results was as follows (Table 1).

The evaluation scale included the following indicators: 0 points—refuses to answer; 1 point—assesses the actions of the children in a particular situation and gives an explanation, even an assessment of the act (behavior) of children in general.

The obtained quantitative and qualitative data give the basis for the conclusion that preschoolers almost without difficulty assess the actions and behavior of children from the situation under discussion (i.e., others): in this column, a sum of 84 points were scored. At the same time, the motivation of actions, i.e., the explanation of why it was necessary to act one way or another (65 points) and the correlation of these actions with the moral norm (63 points), are noticeably lagging behind.

In total, 25% of preschoolers evaded the explanation of why the act can be good or bad, remaining silent or answering “I do not know”. This suggests that preschoolers relate the specific situation to their personal experience, which already has the appropriate assessment by adults or other children. Sometimes, children do not want to give an indirect assessment of their own actions through discussion of situations “with other” children.

It is worth noting the fact that not all young respondents’ specific actions associated with a particular moral norm. Often the assessment of actions in the social and moral situation depended on the subsequent expectations of children, which again came from personal experience. Thus, some of the preschool children, explaining why in one of the situations, a boy had to resort to cheating, said that he “was frightened”, “scared that he’ll be scolded”, “that he will be punished” and so forth. This suggests that the children were already familiar with the consequences of the moral disapproval of these actions. We believe it makes sense to observe these children and their relationships with their parents: are they too strict? Are the age limits of the perception of social and moral norms in preschool age taken into account? etc.

However, there were quite “mature” (for this age) answers: “need to admit” (perfect act), “need to share”, “because girls are always inferior”, “because she is educated” (!), “did a bad thing because you can’t cheat.”

The data of this table show that in general, the social and moral atmosphere in this group was relatively prosperous: more than half of the children were actively involved in conversations of ethical orientation, in general, correctly assessed the social and moral vector of a particular act, and gave it a correct assessment. And this, with appropriate work on the part of the educator, leads to the formation of ideas not just about a particular act, but about the moral norm of behavior adopted in both micro- and macro-society.

At the same time, only two children showed the maximum result (12 points). In two more cases (at the beginning of the list), it was revealed that there were serious problems in social and moral development–they scored only five and six points.

We continued the study, suggesting that the teachers themselves analyze the behavior of their pupils, answering the question “What is a child in relationships with other people?” It was necessary to evaluate the following communicative qualities and types of relations of the child with people: kindness; attentiveness to people; truthfulness and honesty; politeness; sociability; generosity; responsiveness, willingness to help; justice; cheerfulness; responsibility [21].

The use of diagnostic techniques, addressed to both educators and preschoolers, allowed to establish a certain “spread” in the assessment of the children’s behavior.

Thus, according to the results of the first diagnosis, 9% of the children (Respondent 1 and Respondent 2) showed low results in understanding the moral norm. While according to a survey of educators the same X1 was rated a high score on such qualities as generosity, kindness, responsiveness. The question arises: how objective were the teachers in assessing the qualities of this child?

Another group of eight children (36%) was identified who successfully coped with the choice of socially approved behavior. But the same children, judging by a survey of educators, were at a low level of formation of communicative qualities of the person. Again, the question arises – how honest were the educators in assessing the children? Was there any personal interest involved? Were there any manifestations of gender preferences (especially among the eight preschoolers, there were five boys whose behavior was markedly different from that of the girls, who were more socially adapted), etc.?

## 4. Discussion

Thus, the ambiguity of the assessments of the qualitative state of social and moral development of preschool children is revealed. The educators directly involved in the educational process, in daily contact with the children, did not avoid subjectivity in assessing the communicative qualities of the personality of preschool children.

In general, the children, as they should at this age, clearly showed their cheerfulness, responsiveness, responsibility, and generosity. However, unfortunately, the qualities necessary for constructive interaction in social communication such as attentiveness to people, truthfulness, and honesty were the final lines of the quality rating.

The obtained data prove the necessity of purposeful construction of interaction between teachers and children, allowing and taking into account the presence of preschool children’s own judgments, suggestions, and disagreements.

It is known that preschoolers have specific thinking and a little life experience. Therefore, it is necessary to build a strategy of social and moral education of preschoolers based on the accumulation of positive life experience of the child. For example, the provision of mutual assistance (if you help, then you will be helped), the establishment of truthfulness and honesty in the relationship (untruth necessarily someday will be known and they will not believe you), etc. In this case, knowledge should not be reported by the teacher, but should be experienced and understood by each child individually. This feeling will be the basis of unity, harmony, and moral community among children. To the rule, the moral norm children should approach in the process of reasoning. In this case, the norms of morality will be their personal conclusions obtained in the process of practical experience.

You can use a set of cards that demonstrate both the correct behavior (help, compliance, goodwill, approval, joy for another person) and destructive interaction in the children’s team (quarrel, inattention to each other, selfishness, etc.) with the subsequent discussion of these situations and inventing life stories by children [22].

In the process of explaining situations and making up stories, children understand that personal well-being is possible only if the choice is consistent with the well-being of another person. This can be achieved if the relationships with other people are guided by the intentions that you find the best and most suitable for yourself.

Keeping the social and moral vector in the education and development of preschool children, we can recommend educators to develop a program that involves four successive stages:

“It’s Me”: to help the child to form an idea of himself or herself, to identify his or her personal traits, to understand himself or herself as a subject of activity (educational, labor, communicative, etc.).

“Me and You”: to teach a preschooler to build a dialogue communication with a peer (peers), to help to see a friend in the interlocutor, to correct his\her ideas about moral behavior (his\her and others).

“Me and Us”: to expand the child’s understanding of himself\herself as part of the community (group), to identify the comfort zone and the risk zone in communication and interaction with peers, with adults, to adopt the rules of social interaction.

“I and the World”: to lead to the awareness of the involvement of preschool children in the socio-cultural space (kindergarten, school, family, microsocium, etc.), which is very important in the preschool period, to form a readiness for a new social stage-learning at school, to new social contacts, to a new social role, awareness of the value of studying, etc.

This strategy of social and moral education will allow preschoolers to gain experience in understanding themselves and personal characteristics as a result of interaction in the children’s team, experience joy and happiness as a social feeling, develop personal relationships and affection, and strengthen the community of children’s team.

## 5. Conclusions

In conclusion, the following can be noted.

Any science, including pedagogy, develops productively only in the interaction of the relevant theory and practice. The communitarian tradition in the organization of moral development and education of preschool children both in scientific approaches, and in the practice of working with children contributes to the formation of respect for others, friendly interactions with peers, and acceptance and awareness of moral norms and rules governing the behavior and relationships of all subjects of the educational process, etc. It is this orientation that with good reason can be defined as “the area of social and moral development of preschool children”. Research disclaimer: like all psychological and pedagogical research works, this experimental study requires further monitoring; in our case, it is a long-term monitoring of the social and communicative development of preschoolers. The prospects of this scientific work are connected with the adjustment of the criteria of socio-communicative development of preschool children. Furthermore, it is important to work out methodical recommendations for the interaction between teachers and children or between parents and children during social and moral education of the latter. Such methodical recommendations should take into account the children’s own views on moral standards and rules of conduct, and enhance the need for self-discovery in preschoolers, as well as for reflection on and understanding of the social experiences obtained during communication.

## Figures and Tables

**Table 1:** Results of the study of moral concepts of senior preschool age children.

No.	Child under Test	Can Name a Moral Norm				Assessment of Children’s Behaviour				Motivation of Assessment				Result
		No. 1	No. 2	No. 3	No. 4	No. 1	No. 2	No. 3	No. 4	No. 1	No. 2	No. 3	No. 4	
1	Respondent 1				1		1		1		1		1	5
2	Respondent 2	1	1			1	1		1				1	6
3	Respondent 3	1			1	1	1	1	1	1			1	8
4	Respondent 4		1	1	1	1	1	1	1				1	8
5	Respondent 5		1	1	1		1	1	1		1	1		8
6	Respondent 6					1	1	1	1	1	1	1	1	8
7	Respondent 7	1		1	1	1	1	1	1			1	1	9
8	Respondent 8	1	1	1		1	1	1	1	1		1		9
9	Respondent 9	1	1	1		1	1	1	1	1	1	1		10
10	Respondent 10	1	1	1		1	1	1	1		1	1	1	10
11	Respondent 11	1	1			1	1	1	1	1	1	1	1	10
12	Respondent 12	1	1	1	1	1	1	1	1			1	1	10
13	Respondent 13	1		1		1	1	1	1	1	1	1	1	10
14	Respondent 14		1	1	1	1	1	1	1	1	1	1	1	11
15	Respondent 15	1	1	1	1	1	1	1	1	1	1		1	11
16	Respondent 16	1	1	1	1	1	1	1	1	1		1	1	11
17	Respondent 17		1	1	1	1	1	1	1	1	1	1	1	11
18	Respondent 18	1	1	1		1	1	1	1	1	1	1	1	11
19	Respondent 19	1	1	1	1	1	1	1	1	1	1		1	11
20	Respondent 20	1	1	1		1	1	1	1	1	1	1	1	11
21	Respondent 21	1	1	1	1	1	1	1	1	1	1	1	1	12
22	Respondent 22	1	1	1	1	1	1	1	1	1	1	1	1	12
	Σ	63				84				65				
